# Roentgen’s Discovery

**Published:** 1896-03

**Authors:** 


### ROENTGEN'S DISCOVERY.

Our readers are doubtless already informed, through the daily press, of the wonderful discovery of Professor Roentgen, of Würzburg, Germany, whereby photographs may be made of objects through media which are opaque to ordinary light, such, for instance, as wood, ebonite, flesh, cardboard, leather, etc. The apparatus necessary for this process consists, first, of an induction coil, to the two poles of which is attached a Crooke's vacuum tube. It is the radiant energy from this tube that furnishes the powerful "light" necessary for this photography. The photographic plates are inclosed in a wooden plate-holder, which is placed in a horizontal position, and objects to be photographed are interposed between the radiating Crooke's tube and the plate-holder. In this

*Italics ours.

manner good pictures have been obtained of articles inclosed in wooden or cardboard boxes, and of the bones in a human hand. Bone itself is not translucent to this form of light.

One can easily imagine a number of ways by which this discovery may be made applicable in medical and surgical diagnosis. For the location of bullets, to distinguish between dislocations and fractures, to detect exostoses and osteomata, to diagnose a deformed pelvis, to locate foreign bodies in the alimentary tract or abdominal organs, etc. Indeed, it is said that already renal, vesical, and biliary calculi have been discovered by this means, and pistol balls have been located that resisted all other known means of finding them.

The possibilities which lie undeveloped in this discovery, both in physics and medicine, would seem almost without limit. As a means of facilitating precision in diagnosis it will be most welcome in medicine.

---

SIX members of the Georgia Boards of Medical Examiners, who were originally appointed for one year from January 7, 1895, have been reappointed, each for a term of three years. They are Drs. J. B. Baird of Atlanta, and W. A. O'Daniel of Milledgeville, representing the regular school. Drs. R. A. Hicks of Rome, and J. Z. Lawshe of Atlanta, homeopathic; M. T. Salter of Atlanta, and W. V. Robinson of Morgan county, eclectic. Dr. Baird has since been succeeded by Dr. J. C. Olmsted.

---

WE desire to thank our good neighbor, the *Christian Advocate*, for a kind word (February 19): "We have before us the February number of THE ATLANTA MEDICAL AND SURGICAL JOURNAL, published at the Franklin Printing and Publishing Company. A perusal of its contents gives us an appetite for other numbers. It is an able medical journal."

